# Evaluation of BRCAPRO Risk Assessment Model in Patients with Ductal Carcinoma *In situ* Who Underwent Clinical BRCA Genetic Testing

**DOI:** 10.3389/fgene.2016.00071

**Published:** 2016-04-27

**Authors:** Nisreen Elsayegh, Angelica M. Gutierrez Barrera, Kimberly I. Muse, Heather Lin, Henry M. Kuerer, Monica Helm, Jennifer K. Litton, Banu K. Arun

**Affiliations:** ^1^Clinical Cancer Genetics, The University of Texas MD Anderson Cancer CenterHouston, TX, USA; ^2^Departments of Breast Medical Oncology, The University of Texas MD Anderson Cancer CenterHouston, TX, USA; ^3^Biostatistics, The University of Texas MD Anderson Cancer CenterHouston, TX, USA; ^4^Surgical Oncology, The University of Texas MD Anderson Cancer CenterHouston, TX, USA

**Keywords:** ductal carcinoma *in situ* (DCIS), genetics, gene, BRCA1, genes, BRC, BRCA mutation status, BRCAPRO

## Abstract

The authors retrospectively aimed to determine which of the following three scenarios, related to DCIS entry into BRCAPRO, predicted BRCA mutation status more accurately: (1) DCIS as an invasive breast cancer (IBC) entered using the actual age of diagnosis, (2) DCIS as IBC entered with 10 years added to the actual age of diagnosis, and (3) DCIS entered as no cancer. Of the 85 DCIS patients included in the study, 19% (*n* = 16) tested positive for a BRCA mutation, and 81% (*n* = 69) tested negative. DCIS patients who tested positive for a BRCA mutation had a higher BRCAPRO risk estimation (34.61%) than patients who tested negative (11.4%) when DCIS was entered at the actual age of diagnosis. When DCIS was entered with 10 years added to the actual age at diagnosis, the BRCAPRO estimate was still higher amongst BRCA positive patients (25.4%) than BRCA negative patients (7.1%). When DCIS was entered as no cancer, the BRCAPRO estimate remained higher among BRCA positive patients (2.56%) than BRCA negative patents (1.98%). In terms of accuracy of BRCA positivity, there was no statistically significant difference between DCIS at age at diagnosis, DCIS at 10 years later than age at diagnosis, and DCIS entered as no cancer (AUC = 0.77, 0.784, 0.75, respectively: *p* = 0.60). Our results indicate that regardless of entry approach into BRCAPRO, there were no significant differences in predicting BRCA mutation in patients with DCIS.

## Introduction

It has been shown that hereditary predisposition constitutes a major risk factor for the development of breast cancer (BC). Five to ten percent of BC is caused by inheritable gene mutations (Seeber and Driscoll, 2004). The *BRCA1* and *BRCA2* (BRCA) genes account for a high percentage of hereditary breast and ovarian cancer (Seeber and Driscoll, 2004). Carriers of one of the these mutations have a 43–84% risk of developing BC in their lifetime, and up to 65% risk for a contralateral BC (Ford et al., 1998; Chen et al., 2006).

Understanding one's risk for testing positive for a genetic mutation has clinical and personal implications, therefore accurate risk assessment is crucial. Several genetic risk assessment models have been used to predict an individual's likelihood of possessing a BRCA gene mutation. More specifically, the BRCAPRO model is a risk assessment tool part of the CancerGene program. It utilizes Bayes Mendel analysis and ultimately determines those that are at an increased risk of developing breast, ovarian and other cancer types. The BRCAPRO model has been shown to be an accurate model for determining the probability of carrying a genetic mutation (Berry et al., 2002). This model incorporates personal and family history information to determine the probability of a BRCA gene mutation. The analysis incorporates the number of first and second degree relatives along with the tumor histories of the patient and the family members. However, the incorporated tumor history only includes the invasive diagnoses and does not include the pre-invasive lesions.

Ductal carcinoma *in situ* (DCIS) accounts for 25% of all newly diagnosed BC in the United States (American Cancer Society, 2015). It is the most common (80–90%) type of *in situ* carcinoma in the breast, and is a direct precursor to most invasive breast cancer (IBC) (Allred, 2010). Long-term follow-up studies have shown the natural history of untreated DCIS as developing into an IBC (Page et al., 1982, 1995; Eusebi et al., 1994; Erbas et al., 2006). The risk of developing IBC after a diagnosis of DCIS ranges from 14 to 60% at 10 years follow-up (Burstein et al., 2004).

Historically, a diagnosis of DCIS was considered a pre-cancerous lesion and was not entered into the BRCAPRO model (Parmigiani et al., 1998). Currently, DCIS is not specifically accounted for in the BRCAPRO model and there are no standardized guidelines of how DCIS should be entered into the Cancer Gene program. The practice varies among genetic counselors in various parts of the country depending on the clinical training site. Since there no standard guidelines to date, and given the natural course of DCIS progression to IBC in 10 years (Burstein et al., 2004), we used the same criteria established in our institution by adding 10 years to the age of diagnosis (Bayraktar et al., 2012).

It has been previously reported that a BRCA gene mutations have been identified in individuals with DCIS. Hwang et al. reported 37% of individuals with DCIS (with or without IBC) were determined to possess a BRCA gene mutation (Hwang et al., 2007). In addition, Bayraktar et al. identified a 27% BRCA positivity rate amongst individuals with pure DCIS (Bayraktar et al., 2012). Finally, Claus et al. (2005), determined individuals with DCIS had mutation rates similar to those with IBC. Therefore, the different approaches of DCIS entry into the BRCAPRO model may not allow for an accurate risk estimation of a BRCA mutation probability amongst patients with DCIS. Furthermore, recent NCCN Clinical Practice Guidelines In Oncology (NCCN Guidelines®) recommend referral for genetic counseling and consideration of BRCA testing for patients with DCIS; and therefore, accurately assessing these patients for genetic testing has become even more important (NCCN Clinical Practice Guidelines, 2015 ^1^).

In the present study, we aimed to determine which of the following three scenarios, related to DCIS entry into BRCAPRO, predicted BRCA mutation status more accurately: (1) DCIS as an IBC entered using the actual age of diagnosis, (2) DCIS as an IBC entered with 10 years added to the age of diagnosis, and (3) DCIS entered as no cancer.

## Methods

Women with a diagnosis of DCIS who were referred for genetic testing were included in this study. Each participant was either self- or physician-referred to genetic counseling between 2003 and 2011. This study included only patients with DCIS. Any patients with invasive or micro-invasive BC, identified either at time of biopsy or after tumor removal surgery, were excluded. Men were also excluded given that the BRCAPRO model values male BC significantly higher than female BC. Eighty five women were retrospectively identified and received genetic testing. Patients were identified from a prospectively maintained research database, and their characteristics were obtained after institutional review board approval at The University of Texas MD Anderson Cancer Center. All pathologic specimens were reviewed by a dedicated breast pathologist at our institution and all patients underwent routine staging workup.

The likelihood of carrying a BRCA mutation was calculated using The University of Texas Southwestern Medical Center at Dallas CancerGene software, version 5.1 (http://www4.utsouthwestern.edu/breasthealth/cagene/). The BRCAPRO model is a part of the CancerGene software. The BRCAPRO risk is derived through Bayesian probability model taking into account the patients 1st and 2nd degree relatives, age of diagnosis of breast and/or ovarian cancer, ages of unaffected family members (Ready et al., 2009). The mutation frequency and penetrance estimates are derived from BC Linkage Consortium data. Non-carrier incidence rates are derived from SEER data with those with BRCA mutations subtracted out.

BRCAPRO risk estimations were calculated for each patient using the three scenarios. The first estimate was calculated by entering the patient's DCIS as an IBC at their current age of diagnosis. The second estimate was calculated entering DCIS as an IBC with 10 years added to the actual age of diagnosis. The third estimation was calculated with the patient's DCIS entered as no cancer.

Variables entered into the BRCAPRO model for each patient included: gender; current age or age of death; any diagnosis of BC, second primary BC, and ovarian cancer; and age at diagnosis of those cancers; tumor markers (estrogen receptor-ER and progesterone receptor-PR), history of oophorectomy, family history of 1st and 2nd degree relatives with breast and ovarian cancer, race, and Ashkenazi Jewish (AJ) ancestry. For this study, history of oophorectomy was used only if the procedure occurred prior to diagnosis of DCIS. This was to limit identifying those individuals that might have undergone this procedure due to their cancer diagnosis or BRCA mutation status.

Patients were categorized into two groups; age of diagnosis < 40 and ≥40. This was done to account for adding 10 years to the age of diagnosis as a part of our analysis. Within the BRCAPRO calculation, individuals diagnosed with BC age 50 and older are no longer considered to have an early onset of BC.

Entry of information into the BRCAPRO model was standardized and limited to only two study staff members to avoid any discrepancies in how the information was entered. One individual conducted the initial entry and obtained risk estimations. The second staff member performed quality control of the information by entering the variables separately and determined if the same risk estimations were the same. If any information was missing for a patient it was entered into the BRCAPRO model as unknown or not included in the risk assessment.

### Statistical analysis

Two sample *t*-test or analysis of variance (ANOVA) (Snedecor and Cochoran, 1980) were used for the comparison of BRCAPRO between patient groups such as BRCA positive vs. BRCA negative. Three BRCAPRO scores were obtained for each patient as previously defined. The area under the receiver operating characteristic (ROC) curve (AUC) was calculated to evaluate model discrimination (Hosmer and Lemeshow, 2000). A 2-sided *p*-value of 0.05 was considered statistically significant. SAS version 9.2 and S-Plus version 8.04 was used to carry out the computations for all analyses.

## Results

Table 1 outlines demographic and clinical characteristics. Of the 85 DCIS patients included in the study 19% (*n* = 16) tested positive for a BRCA mutation, 81% (*n* = 69) and tested negative. The mean BRCAPRO probability when DCIS was entered at presenting age of diagnosis was 15 (Range = 0–97) vs. 10 (Range = 0–89.9) when calculated 10 years later and 2 (Range = 0–35) when entered as no cancer.

**Table 1:** **Patient characteristics and demographics**.

**Variable**	**Category**	**Counts**	**Percent**
**AVERAGE AGE OF DIAGNOSIS (RANGE) 47 (30–75)**			
BRCA mutation status	Negative	69	81.2
	Positive	16	18.8
Age of diagnosis (by group)	≤40	30	35.3
	>40	55	64.7
BRCAPRO estimations (average)	At age of DCIS diagnosis	0–97	15
	Addition of 10 years to age of DCIS diagnosis	0–89.9	10
	As No Cancer	0–35	2
Race	White	72	84.7
	African–American	3	3.5
	Hispanic	5	5.9
	Asian	5	5.9
Ashkenazi Jewish (AJ) Ancestry	Unknown	1	1.2
	No	77	90.6
	Yes	7	8.2
**FAMILY HISTORY CHARACTERISTICS**			
First degree relatives with BC	0	47	55.3
	1	30	35.3
	2	8	9.4
Second degree relatives with breast cancer	0	28	32.9
	1	37	43.5
	2	20	23.5
Total number of relatives with BC	0	9	10.6
	1	15	17.7
	2	20	23.5
	3	21	24.7
	4	20	23.5
First degree relatives with ovarian cancer	0	80	94.1
	1	5	5.9
Second degree relatives with ovarian cancer	0	77	90.6
	1	6	7.1
	2	2	2.4
Total number of relatives with ovarian cancer	0	68	80.0
	≥1	17	20.0
Bilateral salphingo oophorectomy (BSO)	Unknown	3	3.5
	No	64	75.3
	Yes	18	21.2
**TUMOR CHARACTERISTICS**			
Nuclear grade	I	6	7.1
	II	35	41.2
	III	34	40.0
	Unknown	10	11.8
Estrogen receptor (ER)	Negative	11	12.9
	Positive	59	69.4
	Unknown	15	17.7
Progesteron receptor (PR)	Negative	18	21.2
	Positive	51	60.0
	Unknown	16	18.8

We conducted three separate univariate analyses comparing BRCAPRO at actual age of diagnosis, at 10 years added to the actual age of diagnosis, and DCIS as no cancer. The first univariate analysis (Table 2) comparing BRCAPRO at age of diagnosis by patient characteristics indicated the patients who are BRCA positive had significantly higher BRCAPRO scores at age of diagnosis than those who are BRCA negative (*p* = 0.001). Patients with ER-negative DCIS had higher BRCAPRO scores than those who were ER-positive (*p* = 0.005), and patients with two or more first degree relatives with BC had the highest BRCAPRO scores, compared to those with one or no relatives with BC (*p* = 0.0002). The more relatives with BC, the higher the BRCAPRO scores was when compared to those with less with BC (*p* = 0.00056). AJ ancestry, bilateral salpingo-oophorectomy (BSO), grade, family history of ovarian cancer were not significantly associated with an increased BRCAPRO scores when DCIS was entered at actual age of diagnosis.

**Table 2:** **Comparisons of BRCAPRO at age of diagnosis by patient's characteristics**.

**Variable**	**Group**	***n***	**mean**	**std**	***p* value**
BRCA	Negative	69	11.40	19.40	0.001
	Positive	16	34.61	33.01	
AJ ancestry	No	77	15.7	25.08	0.078
	Yes	7	18.4	12.84	
	Yes	18	19.60	29.84	
First degree relatives with BC	0	47	9.17	18.55	0.0002
	1	30	17.79	22.98	
	2	8	46.91	33.54	
Second degree relatives with BC	0	6	3.97	3.27	0.815
	1	37	15.92	22.3	
	2	20	21.49	31.77	
	Unknown	22	13.53	22.05	
Total relatives with BC	0	9	5.07	6.81	0.0056
	1	15	5.94	11.45	
	2	20	15.40	19.57	
	3	21	16.14	20.19	
	4	20	27.94	24.26	
First degree relatives with ovarian cancer	0	80	16.09	24.57	0.982
	1	5	10.62	16.65	
Second degree relatives with ovarian cancer	1	6	32.42	39.57	0.511
	2	2	50.30	63.78	
Total relatives with ovarian cancer	0	68	12.60	20.60	0.103
	≥1	17	28.44	32.70	
BSO	No	64	13.39	20.62	0.824
	Yes	18	19.60	29.84	
ER status	Negative	11	16.83	26.78	0.005
	Positive	59	12.73	21.56	
PR status	Negative	18	13.79	24.09	0.311
	Positive	51	13.34	22.08	
ER/PR	ER negative-PR negative	11	16.83	26.78	0.098
	ER positive-PR negative	7	9.03	20.16	
	ER positive-PR positive	51	13.34	22.08	
Nuclear grade	I	6	4.58	4.69	0.706
	II	35	13.72	22.97	
	III	34	15.47	21.62	

The second univariate analysis (Table 3) comparing BRCAPRO scores, using DCIS entered with 10 years added to the actual age of diagnosis, by patient's characteristics indicated several significant findings. Consistent with Table 2 results, patients who were BRCA positive had significantly higher BRCAPRO scores than those who were BRCA negative (*p* < 0.0001). Patients with two or more first degree relatives with BC had the highest BRCAPRO scores when 10 years was added to the actual age of diagnosis, compared to those with one or no relatives with BC (*p* = 0.0002). Patients with more relatives with BC had the highest BRCAPRO scores when 10 years was added to the actual age of diagnosis, compared to those with less relatives with BC (*p* = 0.008).

**Table 3:** **Comparisons of BRCAPRO at 10 years later than age of diagnosis by patient's characteristics**.

**Variable**	**Group**	***n***	**mean**	**std**	***p* value**
BRCA	Negative	69	7.09	14.49	<0.0001
	Positive	16	25.38	28.28	
AJ ancestry	No	77	10.66	19.9	0.099
	Yes	7	10.41	8.54	
First degree relative with BC	0	47	5.43	13.9	0.0002
	1	30	12.64	19.31	
	2	8	32.59	28.47	
Second degree relatives with BC	0	6	1.92	1.52	0.796
	1	37	9.39	14.62	
	2	20	15.38	27.05	
	Unknown	22	10.40	19.58	
Total relatives with BC	0	9	2.38	3.09	0.008
	1	15	2.33	3.9	
	2	20	10.63	14.31	
	3	21	12.56	26.59	
	4	20	18.14	22.29	
First degree relative with ovarian cancer	0	80	10.98	19.58	0.750
	1	5	3.32	3.93	
Second degree relatives with ovarian cancer	1	6	24.48	35.23	0.254
	2	2	42.50	54.87	
Total relatives with ovarian cancer	0	68	8.41	15.76	0.096
	≥1	17	19.02	27.89	
BSO	No	64	8.08	15.44	0.553
	Yes	18	14.93	23.3	
ER status	Negative	11	13.96	24.71	0.071
	Positive	59	8.1	15.89	
PR status	Negative	18	10.79	20.98	0.354
	Positive	51	8.50	16.43	
ER/PR	ER negative-PR negative	11	13.96	24.71	0.151
	ER positive-PR negative	7	5.81	13.49	
	ER positive-PR positive	51	8.50	16.43	
Nuclear grade	I	6	2.57	2.73	0.594
	II	35	8.3	17.1	
	III	34	10.69	15.86	

The third univariate analysis (Table 4) comparing BRCAPRO with DCIS entered as no cancer revealed patients who were BRCA positive had significantly higher BRCAPRO scores than those who were BRCA negative (*p* = 0.003). Patients with two or more first degree relatives with BC had the highest BRCAPRO, compared to those with one or no relatives with BC (*p* < 0.0001). Additionally, patients with more relatives with BC had the highest BRCAPRO scores compared to those with less relatives with BC (*p* = 0.0025).

**Table 4:** **Comparisons of BRCAPRO with DCIS entered as no cancer by patient's characteristics**.

**Variable**	**Group**	***n***	**mean**	**std**	***p* value**
BRCA	Negative	69	1.98	6.09	0.003
	Positive	16	2.56	2.63	
AJ ancestry	No	77	2.18	5.87	0.192
	Yes	7	1.34	1.16	
First degree relative with BC	0	47	0.61	1.28	<0.0001
	1	30	2.00	2.94	
	2	8	11.05	14.93	
Second degree relatives with BC	0	6	0.20	0.20	0.429
	1	37	1.73	2.92	
	2	20	2.95	7.63	
	Unknown	22	2.40	7.48	
Total relatives with BC	0	9	0.20	0.21	0.0025
	1	15	0.52	1.19	
	2	20	1.43	2.69	
	3	21	3.17	7.90	
First degree relative with ovarian cancer	0	80	2.15	5.76	0.527
	1	5	1.12	1.38	
Second degree relatives with ovarian cancer	1	6	2.68	3.85	0.366
	2	2	3.10	1.84	
Total relatives with ovarian cancer	0	68	2.05	6.10	0.062
	≥1	17	2.23	2.91	
Bilateral salpingo-oophorectomy (BSO)	No	64	1.75	4.78	0.287
	Yes	18	1.56	2.30	
ER status	Negative	11	0.96	1.83	0.527
	Positive	59	2.29	6.49	
PR status	Negative	18	1.06	2.21	0.330
	Positive	51	2.46	6.90	
ER/PR	ER negative-PR negative	11	0.96	1.83	0.604
	ER positive-PR negative	7	1.20	2.87	
	ER positive-PR positive	51	2.46	6.90	
Nuclear grade	I	6	0.48	0.49	0.679
	II	35	1.93	6.09	
	III	34	2.61	6.24	

**Figure 1:**
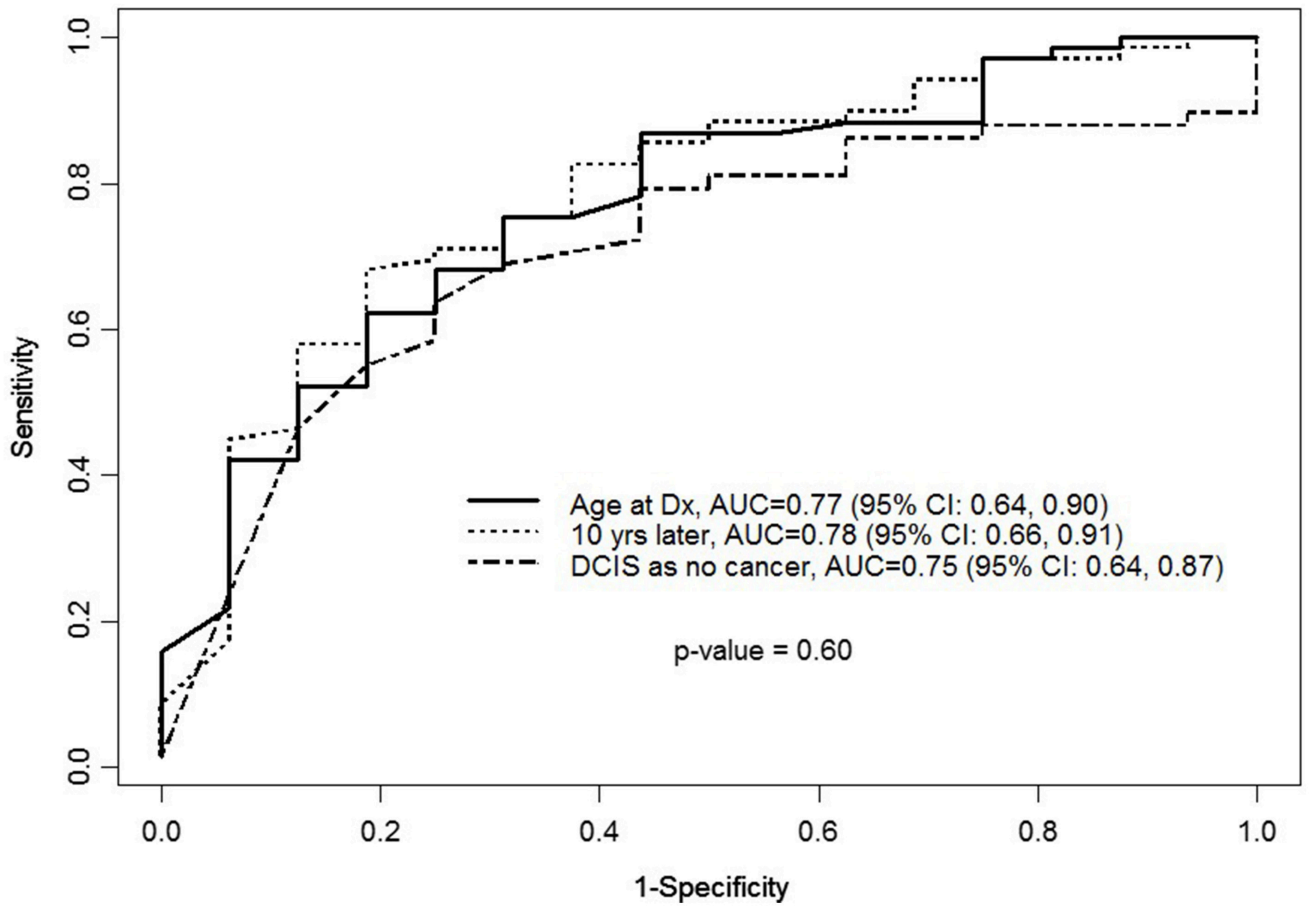
**ROC curve for BRCAPRO with age entered as age of diagnosis or age entered as 10 years older or with DCIS entered as not a cancer in the model when the outcome is BRCA status**.

Finally, for validation we obtained BRCAPRO prediction by calculating area under the receiver operating characteristics (AUC), sensitivity, and specificity. Figure 1 indicates, in terms of accuracy of BRCA positivity, BRCAPRO at age of diagnosis [AUC = 0.77 (95% CI: 0.64–0.90)], BRCAPRO with diagnosis 10 years later [0.78 (95% CI: 0.66–0.91)], and BRCAPRO with DCIS entered as no cancer [0.75 (95 % CI: 0.64–0.87)] were not statically significant (*p* = 0.60) for the comparison of three curves.

## Discussion

The present study determined that patients who are BRCA positive had a higher BRCAPRO score than those who were negative whether DCIS was entered as actual age of diagnosis, or when 10 years was added to the actual age of diagnosis, or as no cancer. To our knowledge, this study uniquely examines the variations of DCIS entry in the BRCAPRO model, and may suggest that regardless of how DCIS is entered, there were no significant differences in predicting BRCA mutation status.

Consistent with our findings, Bayraktar et al. (2012) found among patients with DCIS, those who had a family history of OC or who had BRCAPRO scores ≥10% had a higher rate of BRCA positivity regardless of age at diagnosis. Collectively, these findings suggest that patients with DCIS may be treated similarly to patients with IBC when entered into the BRCAPRO model to determine BRCA mutation probability.

Moreover, Hwang et al. (2007) examined 129 BRCA-positive and 269 BRCA negative women for likelihood of BRCA mutation. BRCA positive patients have an earlier average age of diagnosis for both DCIS and invasive BC than BRCA negative patients. The conclusion of their study reinforces the assertion that DCIS and IBC may treated similarly when using BRCA risk assessments models.

These studies along with our findings, suggest there are no significant differences seen in the BRCAPRO risk estimation when DCIS is entered into the BRCAPRO model of the CancerGene program at current age of diagnosis, or 10 years later, or as no a cancer. Furthermore, the most recent NCCN Guidelines® recommend genetic risk evaluation and genetic testing for individuals with DCIS. This implies that IBC and DCIS may similarly affect the probability of predicting BRCA positivity. The insignificant differences of the AUCs may be due to lack of statistical power despite the observed small differences of the AUCs among the three different scenario. This along with the NCCN guidelines, suggests the need for a study to formally test the hypothesis that these three scenarios predict BRCA positivity similarly. Our rationale for choosing to enter DCIS as invasive cancer includes the followings: (1) when a DCIS patient undergoes genetic counseling, entering the diagnosis as IBC will better assess the risk given the natural progression of the disease, (2) This will help the patient further meet clinical testing guidelines. In this sense, knowing the patient's disease and the BRCA risk assessment will allow for a better chance of receiving genetic testing when deemed clinically necessary. Furthermore, the results will enable patients to take preventive measures when applicable.

Given the diagnosis of DCIS is increasing in the US (Tuttle et al., 2009; Bayraktar et al., 2012), more healthcare providers will be faced with providing accurate genetic risk assessment for BRCA testing for these patients. We believe our study results may provide valuable information to assist in accurate genetic risk assessment for individuals with DCIS, and to help provide a standard entry method into this widely used clinical model.

Since there are no currently established guidelines for entering DCIS, our study suggests that there was no difference between the three approaches evaluated to predict BRCA mutation: (1) DCIS as IBC entered using the actual age of diagnosis, (2) DCIS as an IBC entered with 10 years added to the actual age of diagnosis, or (3) DCIS entered as no cancer. Therefore, heath care providers could safely use the approach closest to their practice.

## Limitations

The study results should be viewed in light of its limitations. This study included patients who were referred for genetic counseling and testing, thus may be not generalizable to all patients with DCIS. When calculating BRCAPRO scores not all patient characteristics were available for every patient. There are also overall limitations of the BRCAPRO model that should be considered. The BRCAPRO model tends to underestimate the importance of ovarian cancer and assumes affected individuals in a family are due to the presence of a BRCA gene mutation. This risk model is a part of the overall genetic assessment, but it certainly is not the only indicator for testing. Other factors such as limited family structure, additional personal risk factors such presence of atypia, and additional family history of other cancers associated with gene mutation as are also considered when deciding for genetic testing but not accounted for in the model. There are other BRCA prediction models used for those with BC that were not utilized for this assessment. Lastly, our sample size was small, which results in a relatively wide 95% confidence interval for the AUCs of the three BRCAPRO scenarios. Future studies are needed to replicate these findings in a larger cohort.

## Conclusion

Our findings suggest that there are no significant changes seen in the BRCAPRO score probability when using the three different scenarios we evaluated. Thus, a diagnosis of DCIS may be treated similar to an IBC diagnosis at current age of diagnosis. It may not be necessary to add 10 years to the patient's age of diagnosis when using the CancerGene Program. The clinical implication of our study is to promote a more consistent and standardized entry method for individuals with a DCIS diagnosis in genetic oncology clinics. Future studies should be done to determine if our outcomes are consistent using other prediction models.

## Author contributions

NE: first author contributed to the majority of the work. AG: Data clean up; analysis, editing. KM: data entry, editing, contributed to some parts of the writing. HL: DATA analysis, and manuscript preparation. HK: Editing. MH: Editing and Final Reading of the article. JL: Editing. BA: Preparation of manuscript, editing.

### Disclosures

The manuscript has never been published and is not under consideration for publication elsewhere. The authors have no financial disclosures to declare.

### Conflict of interest statement

The authors declare that the research was conducted in the absence of any commercial or financial relationships that could be construed as a potential conflict of interest.

## Footnotes

^1^Referenced from the NCCN Clinical Practice Guidelines in Oncology (NCCN Guidelines®). © National Comprehensive Cancer Network, Inc., 2015. All rights reserved. Accessed (May 13, 2015). To view the most recent and complete version of the guideline, go online to www.nccn.org. NATIONAL COMPREHENSIVE CANCER NETWORK®, NCCN®, NCCN GUIDELINES^®^, and all other NCCN Content are trademarks owned by the National Comprehensive Cancer Network, Inc.
